# Wave of single-impulse-stimulated fast initial dip in single vessels of mouse brains imaged by high-speed functional photoacoustic microscopy

**DOI:** 10.1117/1.JBO.25.6.066501

**Published:** 2020-06-11

**Authors:** Yun He, Junhui Shi, Konstantin I. Maslov, Rui Cao, Lihong V. Wang

**Affiliations:** a Washington University in St. Louis, Department of Biomedical Engineering, St. Louis, Missouri, United States; b California Institute of Technology, Caltech Optical Imaging Laboratory, Andrew and Peggy Cherng Department of Medical Engineering, Pasadena, California, United States; c California Institute of Technology, Caltech Optical Imaging Laboratory, Department of Electrical Engineering, Pasadena, California, United States

**Keywords:** photoacoustic microscopy, hemodynamics, blood oxygen saturation

## Abstract

**Significance:** The initial dip in hemoglobin-oxygenation response to stimulations is a spatially confined endogenous indicator that is faster than the blood flow response, making it a desired label-free contrast to map the neural activity. A fundamental question is whether a single-impulse stimulus, much shorter than the response delay, could produce an observable initial dip without repeated stimulation.

**Aim:** To answer this question, we report high-speed functional photoacoustic (PA) microscopy to investigate the initial dip in mouse brains.

**Approach:** We developed a Raman-laser-based dual-wavelength functional PA microscope that can image capillary-level blood oxygenation at a 1-MHz one-dimensional imaging rate. This technology was applied to monitor the hemodynamics of mouse cerebral vasculature after applying an impulse stimulus to the forepaw.

**Results:** We observed a transient initial dip in cerebral microvessels starting as early as 0.13 s after the onset of the stimulus. The initial dip and the subsequent overshoot manifested a wave pattern propagating across different microvascular compartments.

**Conclusions:** We quantified both spatially and temporally the single-impulse-stimulated microvascular hemodynamics in mouse brains at single-vessel resolution. Fast label-free imaging of single-impulse response holds promise for real-time brain–computer interfaces.

## 1 Introduction

Label-free noninvasive functional imaging of the brain is highly desirable.^1^^–^^4^ The most widely used contrast to probe neural activities is the hyperemic response resulted from neurovascular coupling.^4^^–^^6^ However, this process will undergo considerable spatiotemporal broadening from the neuronal activation sites,^7^^,^^8^ making it difficult to interpret and less accurate. The initial dip in hemoglobin oxygenation, due to increased oxygen consumption from activated neurons,^6^^,^^9^^–^^11^ is considered to be a spatially and temporally more confined localizer for the neural activity than the hyperemic response^1^^,^^8^^,^^10^^,^^12^^,^^13^ and has attracted profound interest^1^^,^^6^^,^^8^^–^^18^ since its discovery by optical spectroscopy.^19^ Two label-free imaging techniques, functional magnetic resonance imaging^6^^,^^10^^,^^15^^–^^17^ (fMRI) and wide-field (reflection-mode) optical microscopy,^1^^,^^11^^–^^14^ have both made valuable contributions to the understanding of the initial dip. fMRI, currently the mainstay of neuroimaging, noninvasively obtains cortical-wide mapping of brain function through the detection of paramagnetic deoxy-hemoglobin.^4^^,^^10^ Even in the small-bore form for small-animal imaging, fMRI lacks the spatial resolution to discern the dynamics of cerebral microvessels with diameters <50  μm,^20^ where the initial dip is thought to originate.^8^^,^^10^ Wide-field optical microscopy, in theory, has sufficient spatial resolution, but it suffers from heavy round-trip optical scattering and low sensitivity to small absorption change when resolving deep vessels;^21^ it also lacks depth resolution.^2^ As a result, the initial dip phenomenon is still not fully explored.^6^^,^^12^^,^^15^ Further, we question whether a single-impulse stimulus, much shorter than the response delay, could produce an observable initial dip without repeated stimulation since this is necessary for applications such as the brain–computer interface.

Label-free functional photoacoustic microscopy (fPAM), with optical diffraction-limited spatial resolution, offers unique advantages in detecting hemoglobin response.^5^^,^^22^ Through the photoacoustic (PA) effect,^5^ fPAM ultrasonically detects the absorption of photons from endogenous hemoglobin, thus providing more sensitive detection of hemoglobin oxygenation than scattering-based wide-field optical imaging,^23^ owing to the absorption difference between oxy- and deoxy-hemoglobin molecules. Also since ultrasonic scattering in biological tissues is orders of magnitude weaker than optical scattering,^24^ fPAM can achieve greater penetration depth than wide-field optical microscopy,^5^ enabling imaging of the dynamics of deep cortical microvessels.

To answer our question, we developed Raman-laser-based dual-wavelength fPAM, which spectrally images both hemoglobin oxygen saturation (${\mathrm{sO}}_{2}$) and total hemoglobin concentration (HbT).^25^ Complying with the American National Standards Institute’s safety standard, our fPAM has achieved high-sensitivity volumetric imaging at a 1-MHz one-dimensional (1-D) imaging rate with 2.7-μm lateral and 30-μm axial resolutions, revealing capillary-level vascular dynamics. We imaged the somatosensory area of the mouse cortex and clearly observed a transient wave of the initial ${\mathrm{sO}}_{2}$ dip across individual microvascular compartments, such as arterioles, capillaries, and venules, shortly after applying a single-impulse stimulus.

## 2 Methods

### 2.1 fPAM

**Fig. 1:**
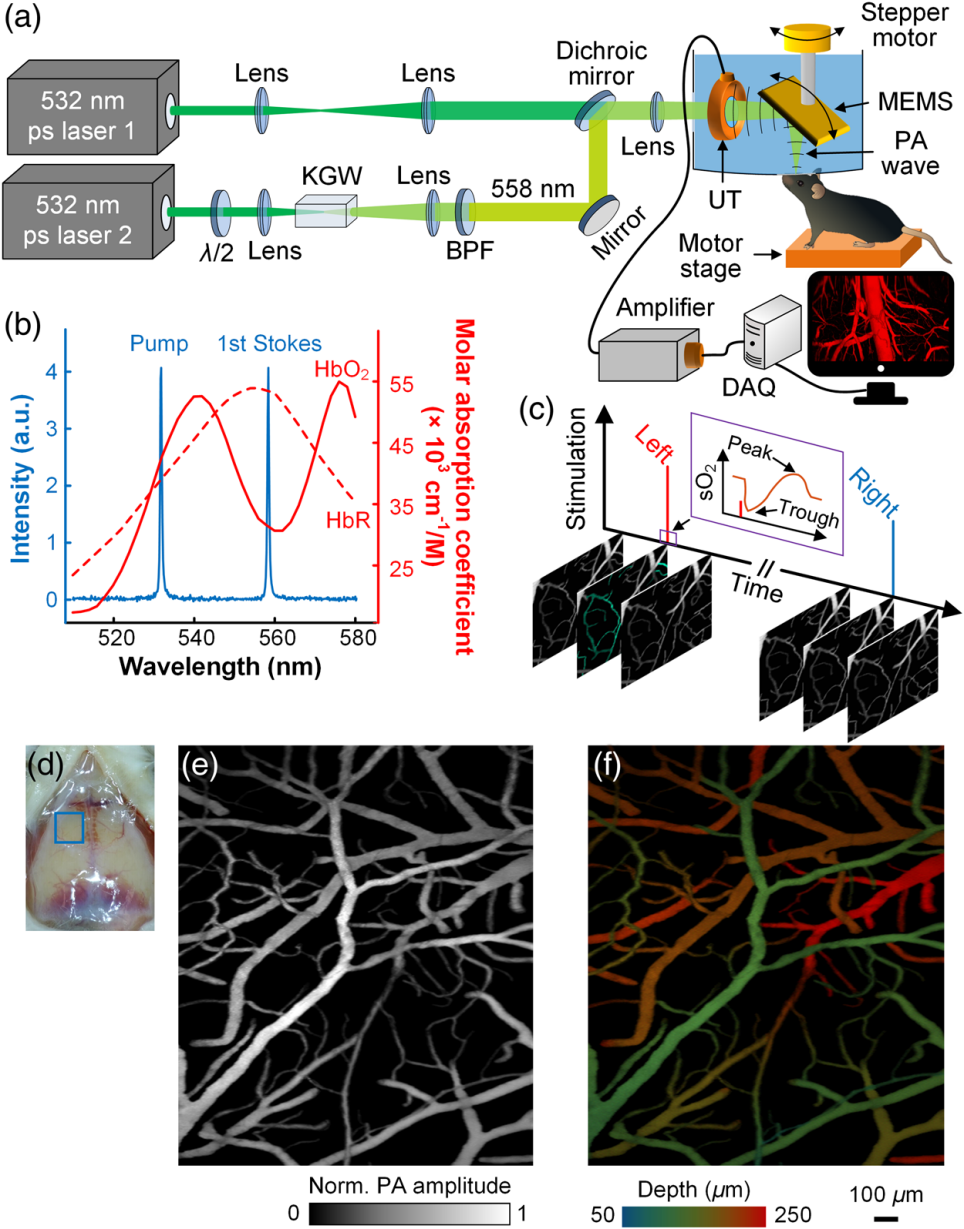
fPAM of the mouse brain. (a) Schematic of the fPAM system: λ/2, half-wave plate; BPF, band-pass filter; DAQ, data acquisition unit; and UT, ultrasonic transducer. (b) Spectrum of the Raman laser plotted on the background of hemoglobin absorption spectra. ${\mathrm{HbO}}_{2}$ and HbR: oxy- and deoxy-hemoglobin, respectively. (c) Scheme for the functional study. Imaging is performed continuously over an area in the somatosensory region illustrated by the blue square in (d), while the mouse receives impulse stimulation to either one of its forepaws alternatingly. The stimulation-induced ${\mathrm{sO}}_{2}$ response starts with a sharp decrease, the initial dip, followed by an overshoot [inset in (c)]. (e) A representative MAP image of the somatosensory region. (f) The depth-encoded image of (e).

Two 532-nm picosecond-pulsed lasers [laser 1 in Fig. 1(a): Olive-1064-4 BW, Huaray Precision Laser; laser 2 in Fig 1(a): APL-4000-1064, Attodyne Lasers] were employed in this dual-wavelength fPAM system, one each for the 558-nm path and the 532-nm path, respectively. In the 558-nm path, the pump laser beam was loosely focused (LA1461-A, Thorlabs; focal length: 250 mm) into the 30-mm-long potassium gadolinium tungstate [$\mathrm{KGd}{\left({\mathrm{WO}}_{4}\right)}_{2}$, KGW] crystal (KGW-702, EKSMA OPTICS; b-cut), and the stimulated Raman scattering (SRS) effect partially converted the 532-nm pump photons to the first Stokes line at 558 nm [Fig. 1(b)]. We employed a picosecond laser here because it provided greater instantaneous power compared with a nanosecond laser of the same pulse energy, making it more easily cause the SRS effect.^26^ Since the Raman gain coefficient of KGW is dependent on the polarization of the pump laser, a zero-order half-wave plate (WPH05M-532, Thorlabs) was used to align the pump laser’s polarization with the a axis of the KGW crystal. The output beam was collimated by another lens and filtered by a band-pass filter (575/25 nm BrightLine, Semrock) to selectively pass the first Stokes line. In the 532-nm path, the laser beam was expanded and collimated by a pair of convex lenses. The 558-and 532-nm laser beams were combined by a dichroic mirror (Dichroic Laser Beam Combiner #86-393, Edmund Optics), focused by an achromatic lens (AC127-025-A-ML, Thorlabs), and reflected into the tissue by the lab-made MEMS scanner. PA waves excited by the laser pulses were also reflected by the MEMS scanner and detected by a polyvinylidene fluoride (PVDF) ultrasonic transducer (custom made by CAPISTRANO LABS: 11-mm diameter, 14-mm focal length, 40-MHz central frequency, 110% −6  dB bandwidth). PVDF transducers match better in acoustic impedance with the tissue coupling water than do ceramic transducers.^27^ Instead of using a complex optical-acoustic splitter, which has a low acoustic transmission efficiency, as in previous PAMs,^22^^,^^28^^,^^29^ we maintained the confocal configuration of the laser beams and the PA waves by passing the laser beams directly through the central hole of this doughnut-shaped PVDF transducer. Both the MEMS scanner and the transducer were immersed in deionized water in a water tank for acoustic coupling. Several critical improvements that have been made since the previous fPAM^22^ are described in the Appendix.

**Fig. 2:**
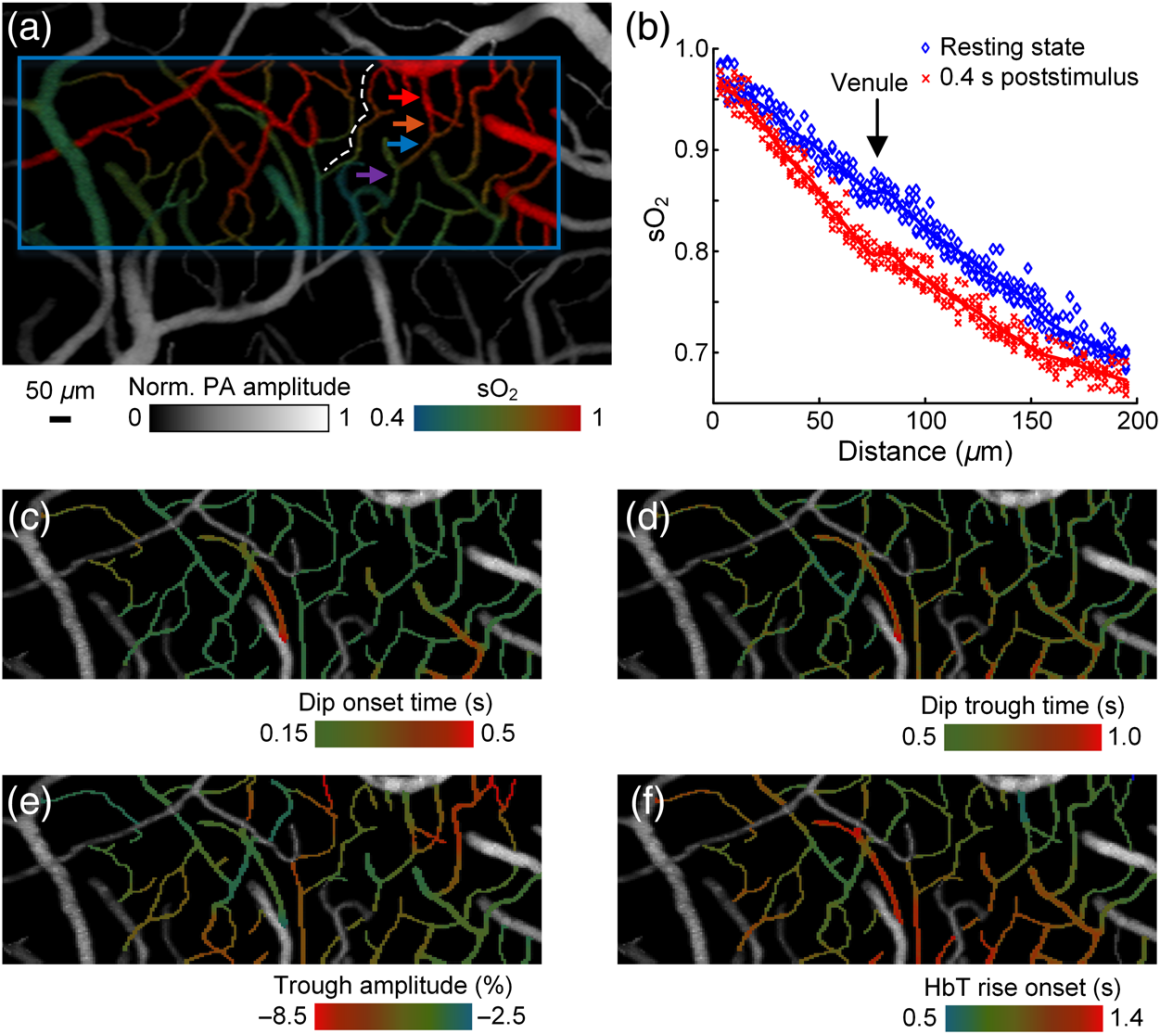
fPAM of cerebral vascular response to electrical stimulations. (a) ${\mathrm{sO}}_{2}$ mapping of the somatosensory area. The blue rectangle illustrates the region of interest for the subsequent functional imaging studies. (b) Oxygen release in resting state (blue) and 0.4 s poststimulusly (red) along a microvessel marked by the white dashed line in (a). It is observed that the capillary had the greatest change between the two states. A plateau, indicated by the arrow, is the site where the blood from another capillary flowed into this vessel. (c) Timing of the initial dip onset, defined by 3σ, superimposed onto the grayscale structure image. (d) Timing of the initial dip trough. (e) Fractional change of the trough of the initial dip. (f) Timing of the hyperemic response onset. Data in (b)–(f) are averaged over five trials.

The PA signals acquired by the transducer were then amplified by a pair of radio frequency amplifiers in tandem (ZFL-500LN+, Mini-Circuits) and digitized by a fast data acquisition unit (ATS9350, Alazar Tech) at a 250-MHz sampling rate. For laser pulse energy calibration, a photodiode (PDA36A, Thorlabs) was used to sample the laser beams, which were split by a wedged optical window (34-245, Edmunds Optics). The MEMS scanner was connected to a stepper motor, and the mouse was supported by a motor-driven translation stage. For raster scanning, fast line scans were performed by the MEMS scanner (Fig. S1 in the Supplementary Material) at a 1-kHz rate (500-Hz resonance frequency), whereas slow orthogonal scans were provided by one of the two motors. The motor stage supporting the mouse was used for the wide-field-of-view (FOV) scanning [Fig. 2(a)], and the stepper motor supporting the MEMS scanner was used for the narrow-FOV scanning [Fig. 2(c)]. Therefore, the mouse remained in a natural motionless state in the latter case.

To separate the two PA signals from the 532- and 558-nm laser pulses, laser 2 was triggered 0.5  μs later than laser 1. This 0.5-μs interval allowed the earlier PA wave to travel ∼0.75  mm (greater than the maximum penetration depth of this fPAM system), and such a short interval also ensured that the two laser beams sufficiently overlapped in the focal plane during scanning.

### 2.2 Data Processing

We employed the Hessian-based vasculature enhancement filter^30^ to extract the blood vessels from our fPAM images. The enhanced image was used to create a binary mask of vessels, which was applied to the raw images to remove nonvessel structures, such as residual blood stains on the brain.

The relative concentrations of oxy- and deoxy-hemoglobin are calculated by spectral analysis of the PA images acquired at 532 nm, a near isosbestic wavelength of hemoglobin, and at 558 nm, a nonisosbestic wavelength [Fig. 1(b)]. Details of this method are described in Ref. 25. In short, the PA signal amplitude is linearly proportional to the local concentrations of oxy- and deoxy-hemoglobins, as described here:

$$[\begin{array}{c} {\mathrm{PA}}_{532\;\mathrm{nm}}/F_{532\;\mathrm{nm}}\\ {\mathrm{PA}}_{558\;\mathrm{nm}}/F_{558\;\mathrm{nm}} \end{array}]K=[\begin{array}{cc} \varepsilon_{\mathrm{HbR}}(532\;\mathrm{nm}) & \varepsilon_{{\mathrm{HbO}}_{2}}(532\;\mathrm{nm})\\ \varepsilon_{\mathrm{HbR}}(558\;\mathrm{nm}) & \varepsilon_{{\mathrm{HbO}}_{2}}(558\;\mathrm{nm}) \end{array}][\begin{array}{c} c_{\mathrm{HbR}}\\ c_{{\mathrm{HbO}}_{2}} \end{array}],$$ (1)

where PA represents the PA signal amplitude, F denotes the local optical fluence, ε is the molar absorption coefficient, c represents the local molar concentration, K is the system’s proportionality coefficient relating the normalized PA signal to the absorption coefficient, and the subscript denotes the wavelength or the form of hemoglobin. Since K is unknown, only relative concentrations can be obtained. Least-squares fitting gives the solution as

$$[\begin{array}{c} c_{\mathrm{HbR}}\\ c_{{\mathrm{HbO}}_{2}} \end{array}]={\left(M^{T}M\right)}^{-1}M^{T}[\begin{array}{c} {\mathrm{PA}}_{532\;\mathrm{nm}}/F_{532\;\mathrm{nm}}\\ {\mathrm{PA}}_{558\;\mathrm{nm}}/F_{558\;\mathrm{nm}} \end{array}]K,\;{\mathrm{sO}}_{2}=\frac{c_{{\mathrm{HbO}}_{2}}}{c_{{\mathrm{HbO}}_{2}}+c_{\mathrm{HbR}}},$$ (2)

where M is the matrix in Eq. (1) and ${\mathrm{sO}}_{2}$ is the oxygen saturation. The system was also calibrated according to Ref. 25.

Fractional changes in HbT are extracted from the images acquired at 532 nm (Fig. S2 in the Supplementary Material). Vessel diameters were measured as the shortest line across a vessel at different angles, with vessel boundaries identified in the unmasked image using the threshold of three standard deviations (3σ) of the background noise.

### 2.3 Experimental Animals

Female Swiss Webster mice (Hsd:ND4, Envigo; 6- to 8-weeks old) were used for the animal experiment. The laboratory animal protocols were approved by the Institutional Animal Care and Use Committee of California Institute of Technology. First, the mouse was anesthetized with isoflurane and then taped to an animal holder with its head tightly fixed by a stereotaxic frame. Its body temperature was kept at 37°C by a heating pad. Second, the scalp was surgically removed, and the skull above the area roughly corresponding to the forepaw of the somatosensory cortex (S1FL region) was carefully thinned to a thickness of ∼50  μm. Ultrasound gel was then applied on the brain to retain moisture and couple the acoustic waves. After the surgery, anesthesia was transferred to α-chloralose by an intraperitoneal injection of a dosage at 50 mg per kg body weight every 2 h. Anesthesia depth was carefully controlled by monitoring the heart rate, respiration rate, and hindpaw pinch reflex. Next, a water tank filled with deionized water was placed on top of the mouse head. The plastic membrane at the bottom of the water tank was in gentle contact with the ultrasound gel applied on the brain. Finally, the mouse was placed under the fPAM for imaging.

### 2.4 Electrical Stimulation Protocol

Electrical stimulations were introduced by two pairs of needle electrodes inserted under the skin of the right and left forepaws. The electrodes were connected to a stimulator (Isolated Pulse Stimulator Model 2100, A-M Systems) for providing the electrical stimuli. A stimulation sequence consisted of a 60-s rest period, a single strong but brief electrical pulse to one of the forepaws, and a 50-s rest period. This electrical stimulus had an amplitude of ∼2  mA and a pulse width of 40 ms [Fig. 1(c)]. Next, the same stimulation was applied to the other forepaw. This sequence was repeated five times, with a 1.0- to 1.5-min gap in between. The stimulation period and intensity were controlled without inducing noticeable motions. We employed a single brief stimulus instead of a pulse train since the initial dip is a fast response that can be confounded by multiple stimuli. Moreover, this stimulation scheme is also used in some other initial dip studies.^14^^,^^31^

## 3 Results

### 3.1 System Performance

First, we characterized the performance of the fPAM system. The 558-nm Raman laser output has a linewidth of ∼0.5  nm [Fig. 1(b)], which was measured by a spectrometer (AvaSpec-ULS2048XL-EVO, AVANTES). And its relative pulse energy deviation was measured to be 1.64%, when producing ∼150  nJ pulses at a 1-MHz pulse repetition rate (Fig. S3 in the Supplementary Material), indicating good monochromaticity and stability. This energy fluctuation, monitored by the photodiode during imaging, was compensated for using Eq. (1) in ${\mathrm{sO}}_{2}$ calculation. Due to the improved sensitivity of our ultrasonic detection scheme, fPAM has achieved a signal-to-noise ratio of 33.2 dB while imaging single RBCs in the cerebral vasculature through a thinned skull (Fig. S4 in the Supplementary Material).

### 3.2 Spatial Profile of the Initial Dip

**Fig. 3:**
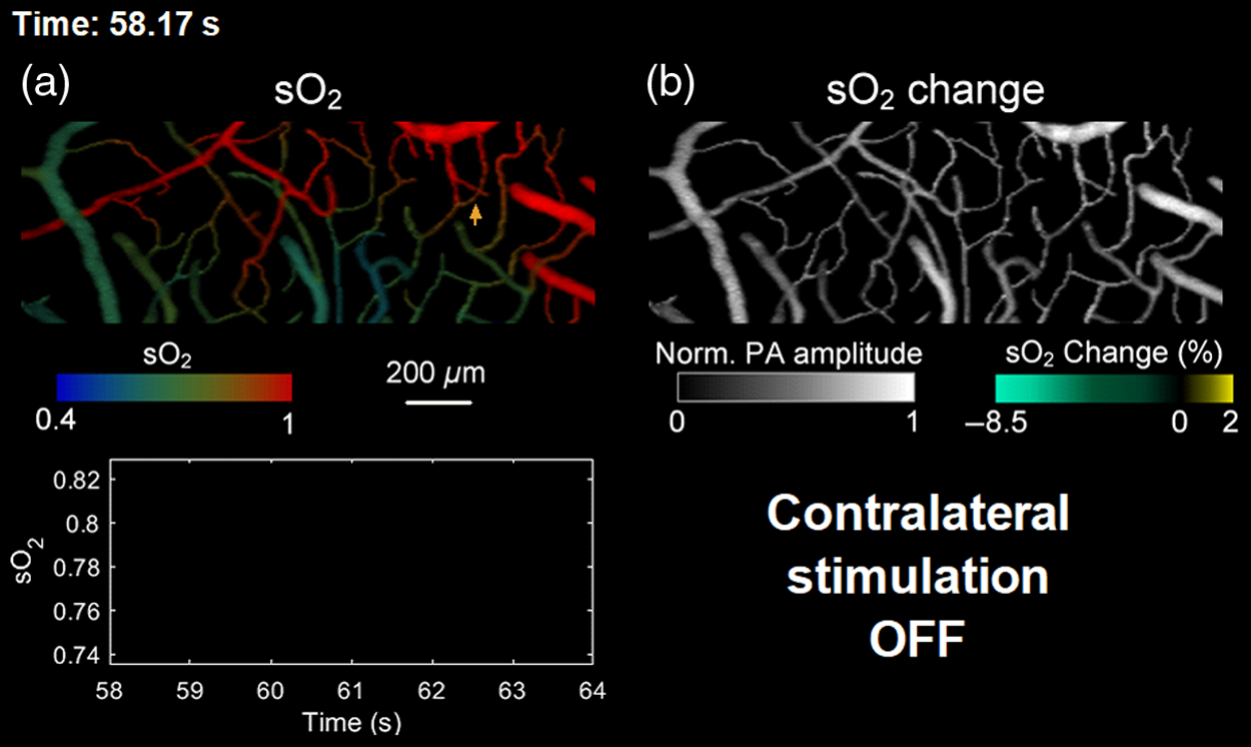
A still image of Video 1 showing the ${\mathrm{sO}}_{2}$ response to the single-impulse stimulation. (a) ${\mathrm{sO}}_{2}$ mapping recorded at 6 Hz and (b) fractional change of ${\mathrm{sO}}_{2}$ superimposed onto the grayscale structural imaging. The curve at the bottom left shows the ${\mathrm{sO}}_{2}$ temporal profile of the area indicated by the arrow in (a) (Video 1, MPEG, 221 kB [URL: https://doi.org/10.1117/1.JBO.25.6.066501.1]).

**Fig. 4:**
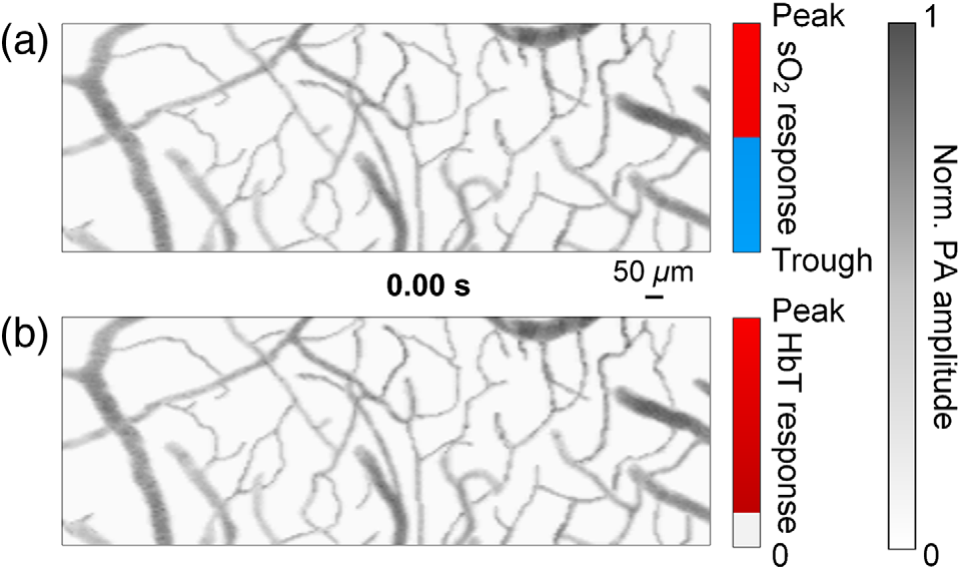
A still image of Video 2 showing the ${\mathrm{sO}}_{2}$ and HbT response to the single-impulse stimulation. The video starts at the beginning of the 40-ms-long impulse stimulus. (a) Response of ${\mathrm{sO}}_{2}$ superimposed onto the structural image. The pseudocolor shows the timing of the trough and peak of the ${\mathrm{sO}}_{2}$ change. (b) Response of HbT. The pseudocolor shows the timing of the peak of the HbT change (Video 2, MPEG, 1.26 MB [URL: https://doi.org/10.1117/1.JBO.25.6.066501.2]).

Using our fPAM, we studied the mouse brain hemoglobin response to a single 40-ms-long impulse stimulus by monitoring both ${\mathrm{sO}}_{2}$ and HbT simultaneously. Here we define impulse as a stimulus with a duration much shorter than the response delay. First, we acquired volumetric images of the somatosensory cortex with an imaging depth of up to 0.5 mm in a single raster scan [Figs. 1(d)–1(f) and Fig. S5, experimental parameters summarized in Table S1 in the Supplementary Material]. The pulse energy used for imaging was ∼150  nJ, which was not strong enough to result in optical absorption saturation.^22^ By fixing the optical focus at ∼200  μm below the skull, the structure and oxygenation of microvessels as deep as layer III of the cortex were clearly mapped [Fig. 2(a)]. In the resting state, the ${\mathrm{sO}}_{2}$ in the microvessels decreased by ∼15% per 100  μm along the vessel [Fig. 2(b)], consistent with literature reports.^32^ Next, a region of interest, illustrated by the blue box in Fig. 2(a), was monitored at a 6-Hz 3-D imaging rate while the mouse received an impulse electrical stimulus at its forepaw. Our high-sensitivity and high-speed fPAM successfully resolved subtle and transient responses from microvessels. Identification of individual mircovascular compartments is shown in Fig. S6 in the Supplementary Material. Upon contralateral stimulation, the ${\mathrm{sO}}_{2}$ from capillaries and venules exhibited a two-phase response: a sharp and short decrease, then a recovery and a small overshoot [Fig. 3]. The fast decrease of ${\mathrm{sO}}_{2}$, the initial dip, started shortly after the stimulus nearly simultaneously across all microvascular compartments [Fig. 2(c) and Fig. S7 in the Supplementary Material], but the trough of the initial dip demonstrated a wave pattern from upstream arterioles propagating through capillaries and further down to venules [Figs. 2(d) and 4]. Similarly, the overshoot from capillaries notably preceded that of the downstream venules. The initial dip at capillaries had a greater fractional change than those from larger vessels [Fig. 2(e)]: ∼8% versus ∼2% to 6%, due to capillaries being the dominant site of oxygen exchange. The trough of the initial dip was followed by the hyperemia resulting from neurovascular coupling, and its spatiotemporal propagation closely resembled that of the overshoot phase in ${\mathrm{sO}}_{2}$ [Figs. 2(f) and 4]. We also observed some arterial dilation co-localized with the hyperemia (Fig. S8 in the Supplementary Material).

### 3.3 Temperal Profile of the Initial Dip

**Fig. 5:**
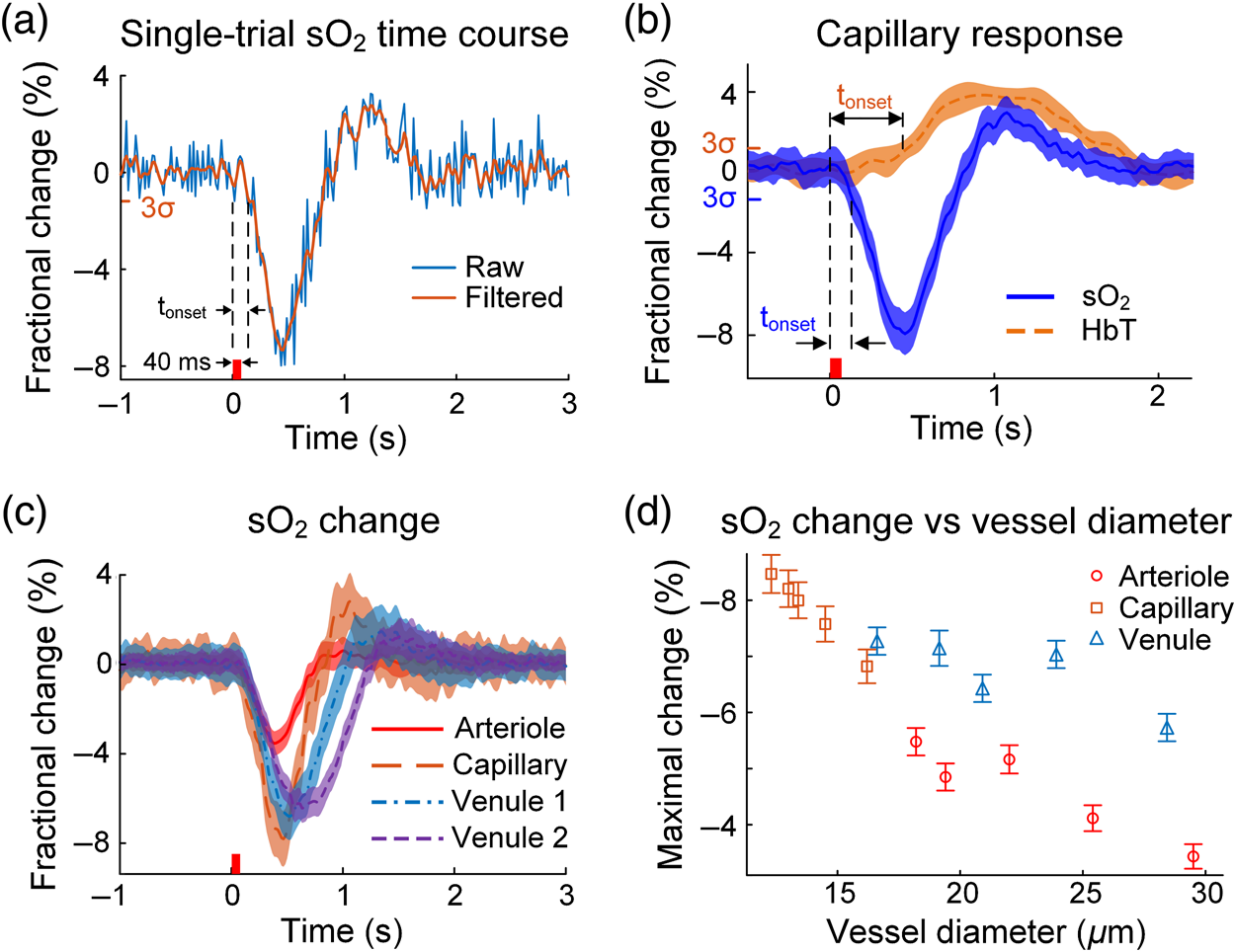
Temporal profiles of the vascular response. (a) A representative single-trial capillary ${\mathrm{sO}}_{2}$ time course in response to an impulse stimulus. The raw data (blue line) is filtered with a Bessel low-pass filter (25-Hz cutoff frequency) to produce the orange curve. We chose the Bessel filter because it preserves the wave shape of the filtered signal in the passband and therefore maintains causality. (b) Comparison of the averaged ${\mathrm{sO}}_{2}$ and HbT time courses at the capillaries. By applying the threshold of 3σ, the onset times of the ${\mathrm{sO}}_{2}$ and HbT responses are calculated as 0.13 and 0.46 s, respectively. (c) Time courses of the ${\mathrm{sO}}_{2}$ fractional changes in four vessel segments representative of the different microvascular compartments. An example of these representative segments is illustrated by the color-coded arrows in Fig. 2(a). (d) Initial dip amplitude versus vessel diameter for arterioles, capillaries, and postcapillary venules. Data in (b) and (c) are averaged over five trials on each of the five mice; data in (d) are averaged over five trials; the stimulus is illustrated by the small red bar on the horizontal axis; venule 1 and venule 2 denote postcapillary and second-stage venules, respectively; error bar shows standard error.

Next, we performed fast line scans (1 kHz) for 2-D imaging across representative vessel segments from different microvascular compartments [arrows in Fig. 2(a)] to acquire finer temporal profiles of the impulse response (Fig. 5). Figure 5(a) shows a representative single-trial ${\mathrm{sO}}_{2}$ response to an impulse somatic stimulation. The initial dip started first in capillaries [Figs. 5(b) and 5(c)], with the onset time averaged over five mice being 0.13±0.01  s (all time points are relative to the beginning of the stimulus). The initial dip onset in other microvascular compartments occurred only up to 0.05 s later than that in capillaries, and it reached the trough at 0.4 to 0.7 s, with the trough from venules arriving ∼0.3  s later than that from arterioles [Fig. 5(c)]. The capillary has a trough fractional change of up to 8%, which is more than twice that of the arteriole but only ∼20% greater than that of the postcapillary venules [Fig. 5(d)]. Second-stage venules, the venules joined by postcapillary venules, also had an initial dip with a significantly longer duration than capillaries and arterioles, probably due to the out-of-phase influx of deoxygenated blood from different capillaries. Following the trough, the ${\mathrm{sO}}_{2}$ levels quickly recovered, and capillaries and venules manifested a weak and flat overshoot, which was insignificant in arterioles [Fig. 5(c)]. This overshoot phase lasted up to 2 s in venules. The hyperemia started first at the arteriole around 0.4 s, slightly ahead of the trough of the initial ${\mathrm{sO}}_{2}$ dip in all compartments of the vessel [Fig. 5(b) and Fig. S9 in the Supplementary Material]. Its peak amplitudes at the arteriole and capillary were similar, while venules had a weaker response but of a longer duration. The tail of hyperemia closely coincided with that of the overshoot phase of ${\mathrm{sO}}_{2}$ response [Fig. 5(b)], indicating a strong relation between them.

Meanwhile, the ipsilateral stimulation resulted in only a hyperoxic response in ${\mathrm{sO}}_{2}$ and a hyperemia of a smaller amplitude, which occurred at a time point similar to that resulting from contralateral stimulations (Fig. S10 in the Supplementary Material).

## 4 Discussion

Since functional imaging often indirectly probes neural activities through measurement of hemodynamics, correct interpretation of this contrast is crucial. Considerable research has been devoted to the initial dip, which is thought to be directly related to the local neuronal metabolism. However, despite being frequently observed and extensively studied by various imaging/spectroscopy modalities, its spatiotemporal dynamics in individual microvascular compartments remain inconclusive. It was unclear whether a single-impulse stimulus could produce an observable initial dip. Enabled by advances in sensitivity and imaging speed, our fPAM system imaged single-impulse-stimulated hemodynamics, which provides new insight into the initial dip phenomenon at high spatial and temporal resolutions. fPAM data showed that the initial ${\mathrm{sO}}_{2}$ dip started as early as 0.13 s after the onset of the stimulus. Considering a typical value of $∼4\times 10^{-5}\;{\mathrm{cm}}^{2}/s$ for the oxygen diffusion constant in tissue,^33^ an extravascular hypoxic gradient would travel ∼33  μm in 0.13 s, which is consistent with the general capillary density.^34^

A propagation of the initial dip across different microvascular compartments was also observed. The initial dip amplitude in our data was notably higher than previous reports. The reason could be that our study was on a single-microvessel basis, while others were based on averaging over considerably larger voxels. The choice of anesthetic and the depth of anesthesia may also affect the amplitude of hemodynamic responses.^35^

Our motivation for developing a Raman laser was the limited choices of wavelengths for high-repetition-rate lasers.^26^^,^^36^ SRS effectively shifts the laser wavelength with a conversion efficiency usually one order of magnitude higher than commercial optical parametric oscillator systems.^37^ Compared with other popular Raman materials, KGW possesses many desirable features,^38^ such as convenient operation, a high Raman gain coefficient, a high thermal damage threshold, a high thermal conductivity, and low thermal lensing, of which the last two factors are most critical for developing stable high-repetition-rate Raman lasers. Our free-space Raman laser also achieves better monochromaticity than fiber-based versions.^39^^,^^40^ In addition, the free-space design makes it easier to switch to other Raman materials, such as $\mathrm{Ba}({\mathrm{NO}}_{3}{)}_{2}$, adding more wavelength choices. The ultralow SRS threshold of KGW also enables the system to conduct simultaneous imaging at more than two wavelengths by employing additional higher-order Stokes and anti-Stokes lines. For example, melanoma progression and its hypoxia environment can be monitored by our fPAM working simultaneously at 532, 558, and 658 nm (fourth Stokes line), further extending the scope of this study.

In summary, we developed a dual-wavelength fPAM based on a Raman laser at a 1-MHz 1-D imaging rate. Through direct imaging of oxygenation in individual microvascular compartments with optical absorption-based contrast and high spatiotemporal resolution, fPAM has achieved exquisite sensitivity for hemoglobin imaging. *In vivo* experiments quantified the single-impulse-stimulated initial dip from cerebral microvessels, providing new insights into this elusive phenomenon. Translation to the brain–computer interface could be a future exploration for fPAM. Although advances in fPAM have opened avenues for *in vivo* biomedical studies, at present, our fPAM can only image anesthetized animals. Imaging awake animals with a head-mount fPAM is worth pursuing. Moreover, fPAM still cannot image all of the cerebral vessels due to insufficient resolution and the limited-view issue.^41^ Innovation on the optical illumination and ultrasonic detection designs to address this limitation is also an important future exploration.

## 5 Appendix: Innovation Since the Previous fPAM

In our previous fPAM,^22^ the confocal alignment of the laser beams and the PA waves was achieved through an optical-acoustic splitter, which had a low efficiency for both optical and acoustic transmissions. Here, we circumvent this issue by employing a PVDF transducer with a hollow core for direct laser passage. The acoustic impedance of PVDF matches better with the tissue-coupling water than the lead zirconate titanate (PZT) ceramic transducer used in the previous version, which improves overall acoustic transmission efficiency.^27^ This setup achieved a ∼50% increase in detection sensitivity despite the lower sensitivity of PVDF. Further, PVDF transducers often offer a broader bandwidth than PZT transducers, improving the axial resolution of PAM.

The ${\mathrm{sO}}_{2}$ measurement in the previous work was based on optical absorption saturation, thus requiring a laser exposure exceeding the ANSI safety limit. Here, we employ the more established spectroscopic method^42^ for measuring ${\mathrm{sO}}_{2}$. For *in vivo* brain imaging, the laser exposure on the tissue surface is $18\;{\mathrm{mJ}/\mathrm{cm}}^{2}$, within the ANSI standard, making it safe for future human imaging. Unlike several previously reported systems in which the scanners were made of proprietary materials or required expensive machining equipment,^22^^,^^43^^–^^45^ the MEMS scanner that we developed here was fabricated entirely from commercially available parts using common engineering tools, facilitating replication. We also achieved a fivefold increase in imaging speed.

In summary, compared with the previous fPAM, our system’s detection sensitivity is significantly improved due to the low acoustic signal loss of the PVDF transducer. The spectroscopic method employed here for the ${\mathrm{sO}}_{2}$ measurement does not require optical absorption saturation. Therefore, our ${\mathrm{sO}}_{2}$ mapping is also more robust, especially for deep vessels where the light attenuation is too strong to safely satisfy optical absorption saturation. We expect our fPAM to achieve a penetration capability similar to the previous one. However, a rigorous study is beyond the scope of this report.
