# Supplementary material for: Wave of single-impulse-stimulated fast initial dip in single vessels of mouse brains imaged by high-speed functional photoacoustic microscopy
Source: J Biomed Opt. 2020 Jun 11;25(6):066501. doi: 10.1117/1.JBO.25.6.066501 (PMC7289453; doi:10.1117/1.JBO.25.6.066501)
Supplement: Supplementary file 1 [file JBO_025_066501_SD001.docx]

**Supplementary Material**

**Table S1. Summary of fPAM parameters for mouse brain imaging**

| Parameters | Fig. 1e | Fig. 2c |
| --- | --- | --- |
| Field of view (*x* × *y* mm^2^) | 2.2 × 2.0 | 0.6 × 1.8 |
| Choice of *x* scanning | Motor stage supporting  the mouse | Stepper motor connected to the MEMS scanner |
| Choice of *y* scanning | MEMS scanner | MEMS scanner |
| 3D imaging rate (Hz) | 1.5 | 6 |


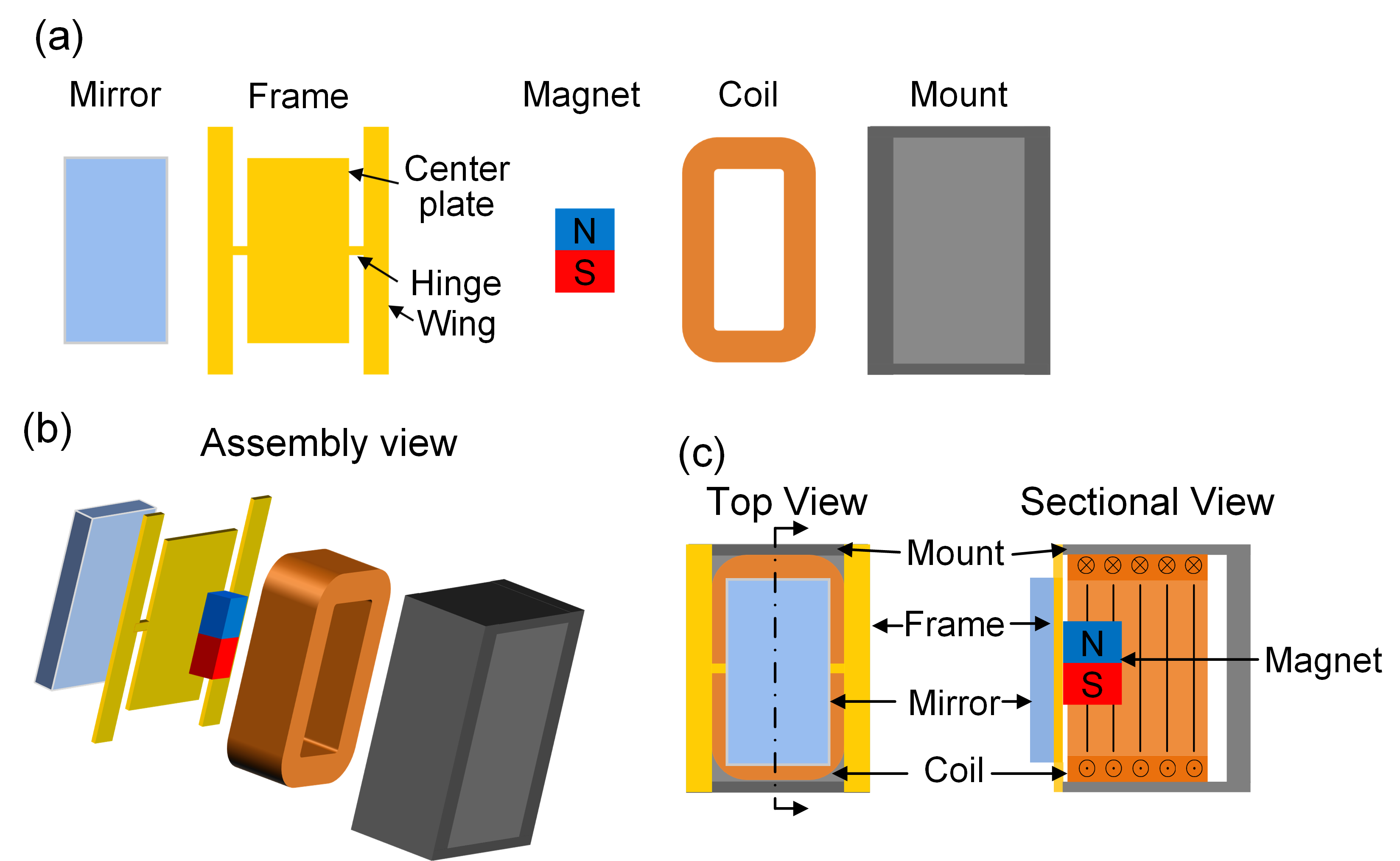


**Fig. S1** Schematic of the MEMS scanner. (a) Breakdown of the MEMS scanner. The scanning part of the scanner is comprised of the mirror, the center plate of the frame, and the permanent magnet. The stationary part is comprised of the two wings of the frame, the inductor coil, and the mount. The scanning part is connected to the stationary part via the two hinges of the frame. (b) Assembly view of the MEMS scanner. (c) Top and sectional views of the finished MEMS scanner. The actuation force is generated by the electromagnetic interaction between the electrical current through the inductor coil and the permanent magnet.


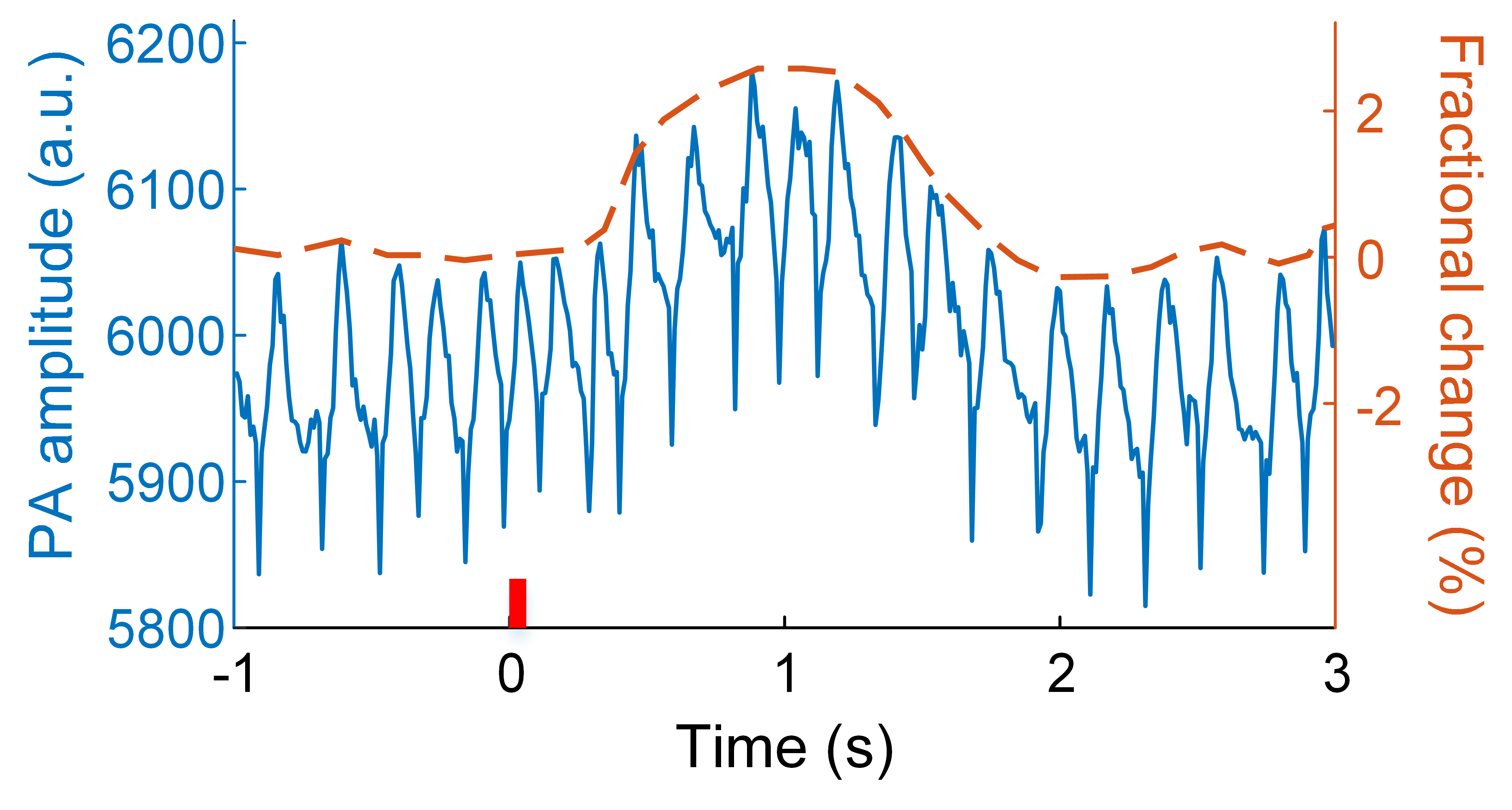


**Fig. S2** Extraction of total hemoglobin concentration changes**.** The blue line is the raw signal from line scans with the 532 nm laser. The fractional change, shown by the dashed line, is obtained by filtering out the heartbeat dynamics with low-pass filters. The red bar on the horizontal axis denotes the stimulus instant.


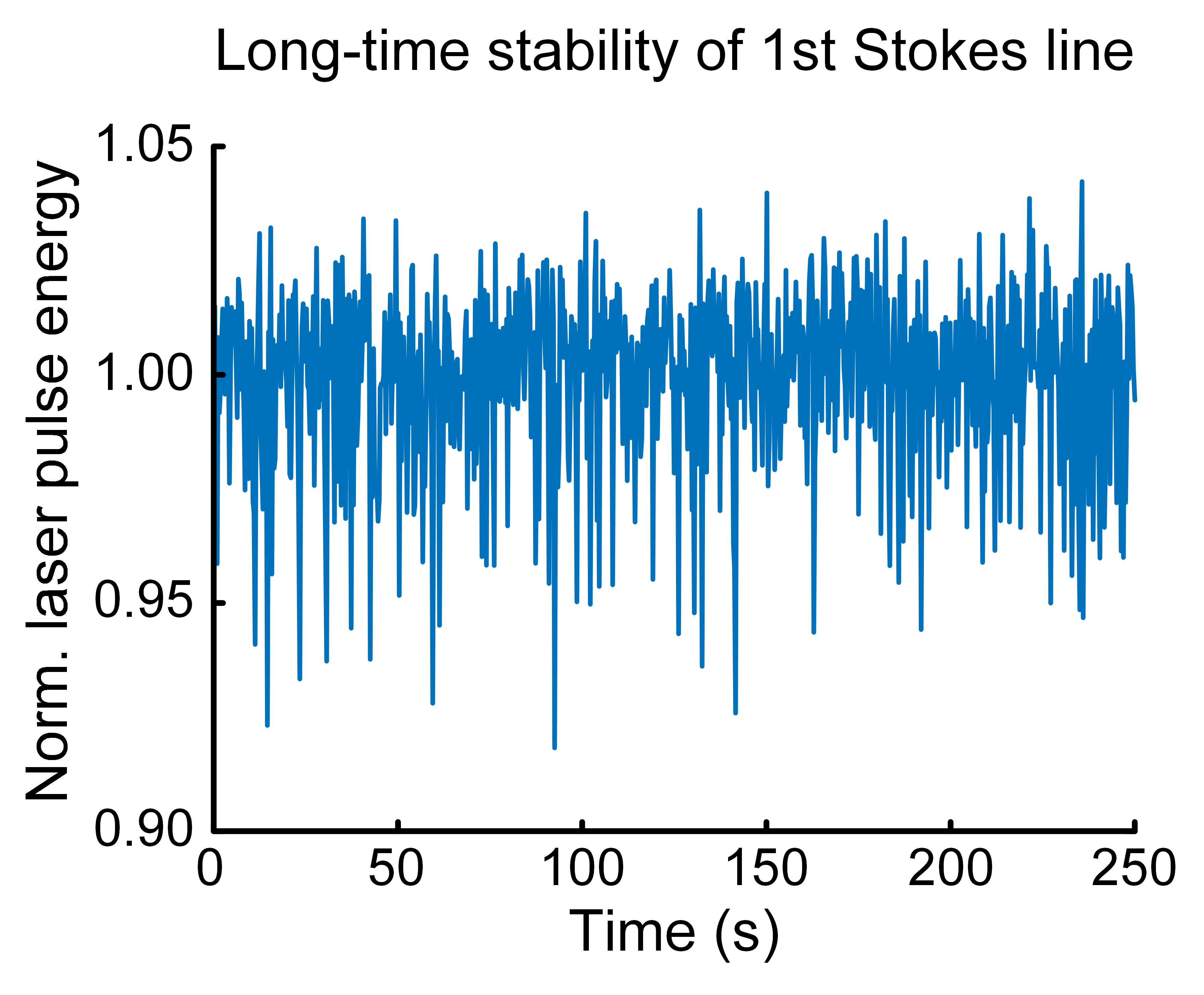


**Fig. S3 Stability test of the 558 nm first Stokes line output.** The laser pulse energy was sampled by a high-speed photodiode (DET10A, Thorlabs).


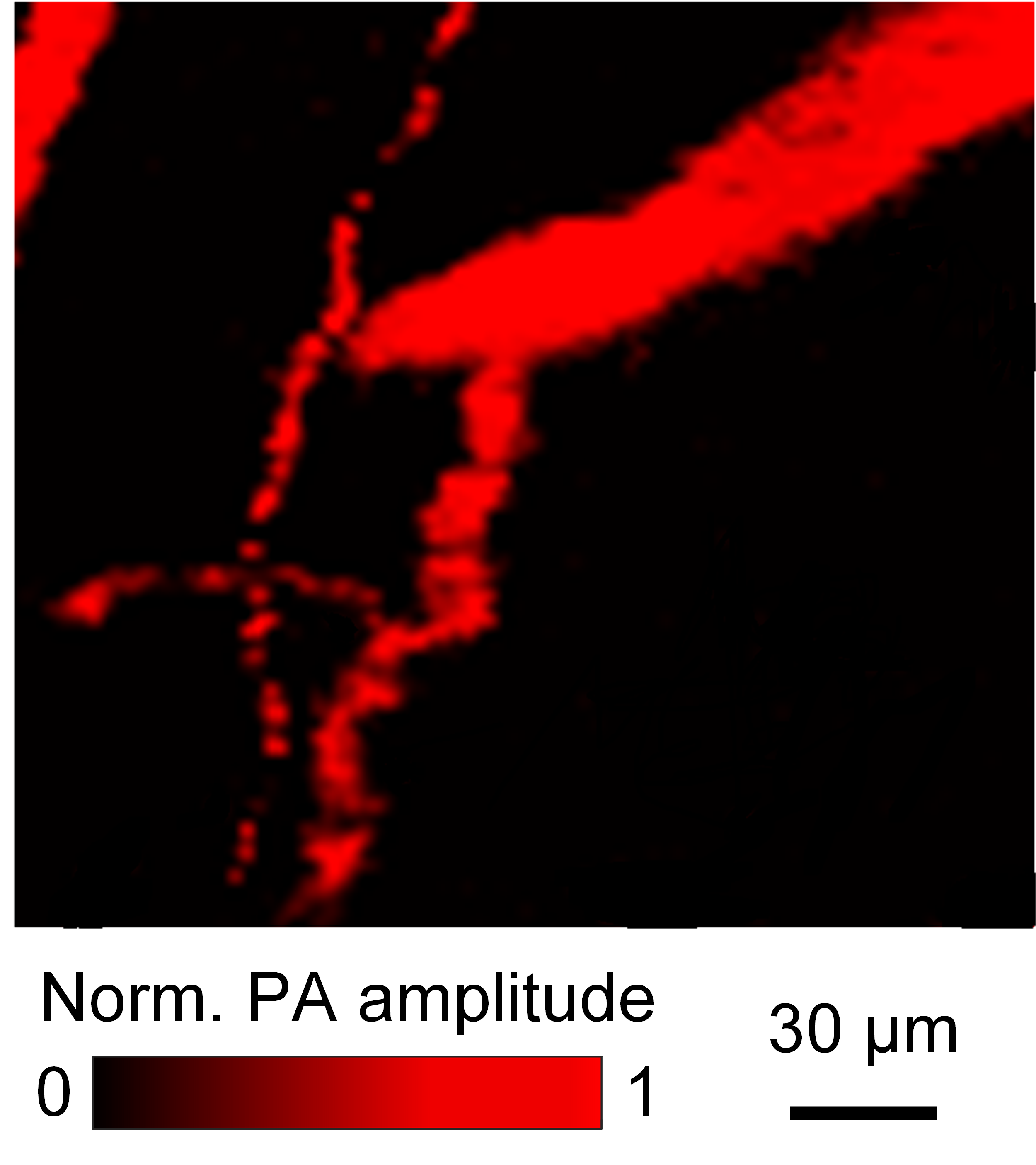


**Fig. S4.** Imaging of cerebral blood vessels, showing individual RBCs. In this experiment, the mouse brain was pressed firmly against the membrane at the bottom of the water tank to facilitate discontinuous flows of RBCs.


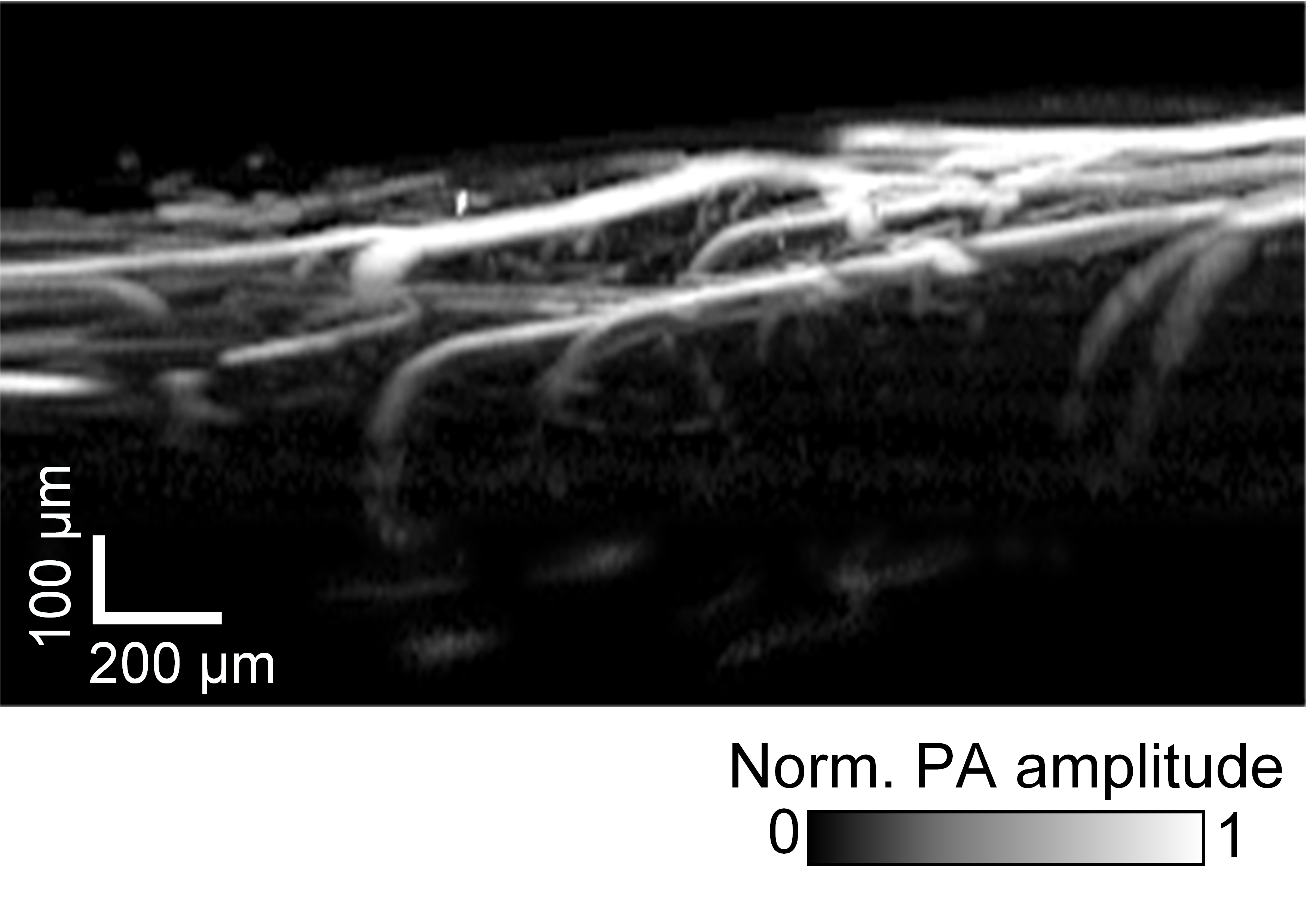


**Fig. S5** Representative PA image of cerebral vasculature projected onto the coronal plane. Volumetric data is obtained by single-plane scanning with the depth axis resolved by the times of flight of PA signals.


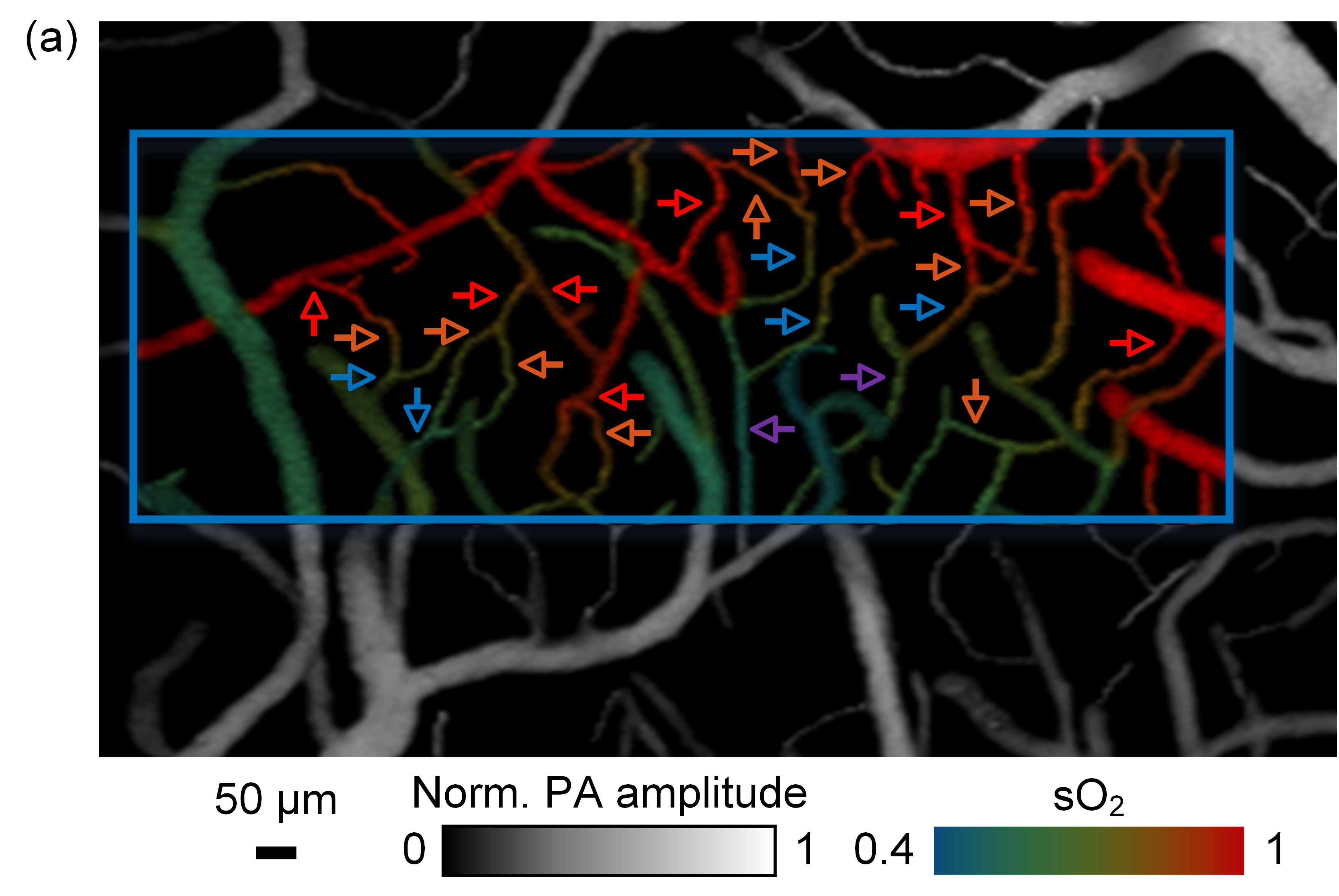


**Fig. S6** Example of microvascular compartment classification. Arterioles, capillaries, postcapillary venules and second-stage venules included in the statistical analysis are labeled with red, orange, blue and purple arrows, respectively.

**
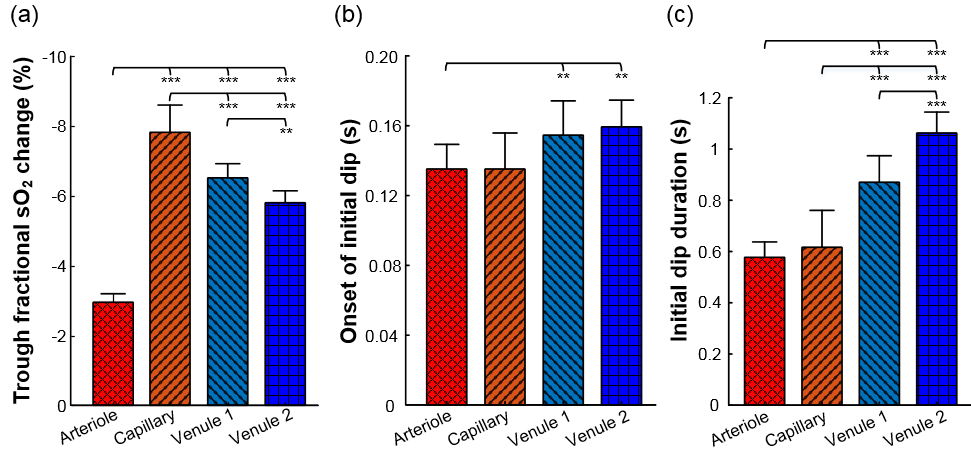
**

**Fig. S7** Statistical analysis of the hemodynamics in four vessel segments representative of the different microvascular compartments. Venule 1 and venule 2 denote postcapillary and second-stage venules, respectively. Data are averaged over five trials on each of the five mice; error bar, standard error; statistics, paired Student’s *t*-test; *P* values, *** < 0.001; ** < 0.05.


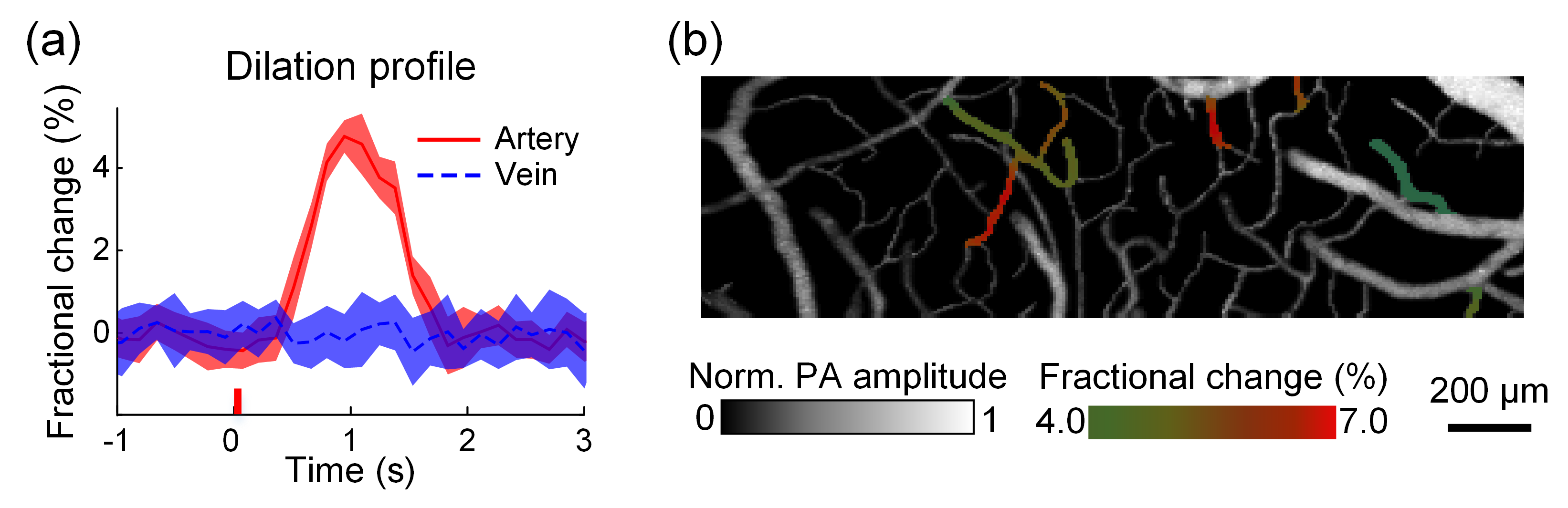


**Fig. S8** Profile of the vessel dilation response. (a) Time course of the vessel diameter change in arteries (red) and veins (blue). The arterial curve is averaged over the arteries detected with vasodilation, and the venous curve is averaged over the veins of similar diameters as those arteries; the error bars denote standard deviations. (b) Mapping of the peak amplitude of vessel dilation. Diameter changes in smaller vessels were not resolved. Data were averaged over five trials.

**
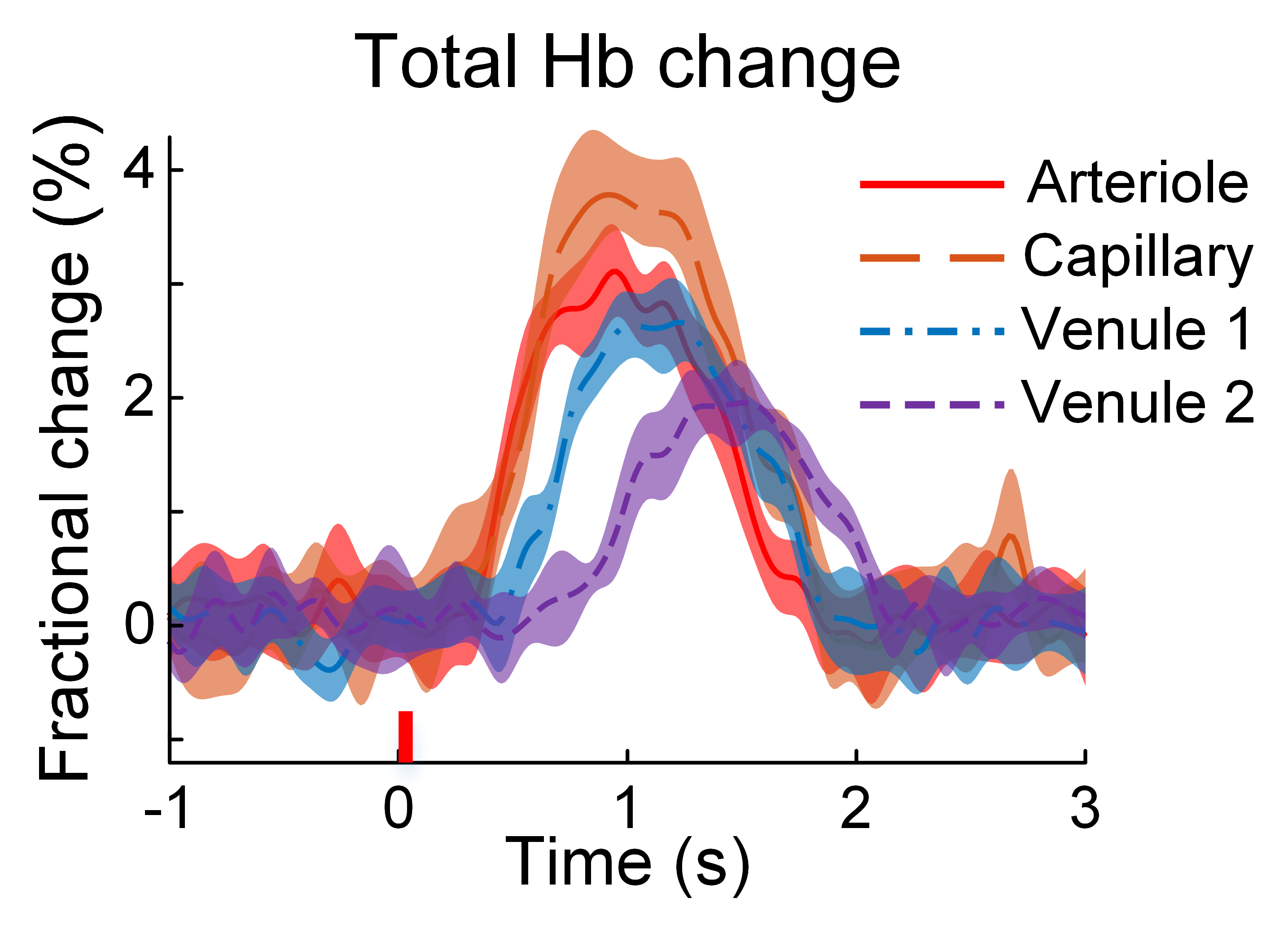
**

**Fig. S9** Time courses of the HbT fractional changes in four vessel segments representative of the different microvascular compartments. An example of these four stages is illustrated by the arrows in Fig. 2a. Data are averaged over five trials on each of the five mice; the stimulus is illustrated by the small red bar on the horizontal axis; error bars show standard errors.

**
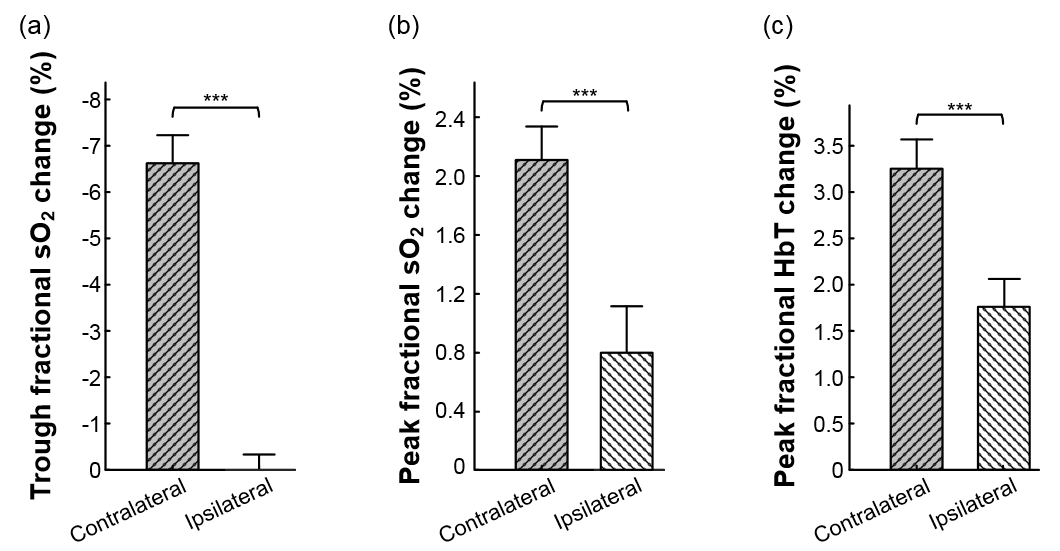
**

**Fig. S10** Comparison of vascular responses in the core response areas. (a) Trough amplitude of initial dips from contralateral and ipsilateral stimulations. (b) Peak value of the sO_2_ overshoot. (c) Peak value of the hyperemic response. Data are averaged over five trials on each of the five mice; error bar, standard error; statistics, paired Student’s *t*-test; *P* values, *** < 0.001.
